# Drug poisoning and associated factors in western Saudi Arabia: A five-year retrospective chart review (2011–2016)

**DOI:** 10.12669/pjms.335.13119

**Published:** 2017

**Authors:** Sami H. Alzahrani, Ali H. Alqahtani, Fayssal Mostafa Farahat, Mohammed Abdel Galil Elnour, Jamil Bashawri

**Affiliations:** 1 Sami H. Alzahrani, MD, Department of Family and Community Medicine, Faculty of Medicine, King Abdulaziz University, Jeddah, Saudi Arabia; 2 Ali H. Alqahtani, MD, Public Health Administration, Ministry of Health, Jeddah, Saudi Arabia; 3 Fayssal Mostafa Farahat, MD, MSc, Ph.D., Department of Public Health and Community Medicine, King Saud bin Abdulaziz University for Health Sciences, King Abdullah International Medical Research Center, King Abdulaziz Medical City, Jeddah, Saudi Arabia; Menoufia University, Egypt; 4 Mohammed Abdel Galil Elnour, MD, MPH, Public Health Administration, Ministry of Health, Jeddah, Saudi Arabia; 5 Jamil Bashawri, MD, SBFM, Department of Family and Community Medicine, Faculty of Medicine, King Abdulaziz University, Jeddah, Saudi Arabia

**Keywords:** Drug poisoning, Prevalence, Pattern, Jeddah

## Abstract

**Objective:**

Drug poisoning is a globally common cause of emergency-room admissions. This study explores drug-poisoning prevalence patterns, associated risk factors (gender, age and exposure circumstances), and outcomes in western Saudi Arabia.

**Methods:**

Retrospective analysis of Clinical drug poisoning cases (2011-2016). The data were retrieved from the Saudi Ministry of Health’s record and Patients’ medical charts were analyzed.

**Results:**

The Ministry of Health received 1,474 reports of drug poisoning during 2011–2016. More than half involved females (n=885, 60%) or young children (0-4 years old) (n=764, 51.8%) and occurred accidentally (n=786, 53.3%); almost all had an oral route of poisoning (n=1,466, 99.5%). The cases most frequently involved analgesic and non-steroidal anti-inflammatory drugs (n=373, 25.2%); antiepileptic, antipsychotic, psychoactive, and anxiolytic drugs (n=229, 16.3%); antihistamine, asthma, flu, and cough drugs (n=157, 12.0%); and antibiotic, anti-fungal; and antiprotozoal drugs (n=74, 5.0%). Antidotes were administered in only 2.2% of cases, and no deaths were reported.

**Conclusion:**

The drug poisoning cases involved females and young children (younger than 5 years old) and the most cases were accidental, and the most commonly used drugs were analgesics (Panadol), followed by antipsychotics, antihistamines, and antiepileptics (Tegretol).

## INTRODUCTION

Over the past four decades, the field of pharmacy and drug therapy has seen a lot of developments, which have resulted in significant improvements in patient care, but at the same time, an increasing rate of drug-related problems accounts for an estimated 5%-10% of hospital admissions.^1^-^3^ A drug-related problem is defined as “an event or circumstance involving drug therapy that actually or potentially interferes with desired health outcomes.”^1^,^2^,^4^ Major drug-related problems include drug overdose and poisoning, which have started to attract international concern due to rising mortality and morbidity rates.^5^ Several studies have reported that drug poisoning is a major cause of emergency department (ER) admissions, with various incidences across countries, regions, socioeconomic status, and cultures.^2^,^5^-^7^ In the United States, almost 1.1 million patients were admitted to ERs for drug poisoning over a three-years period, resulting in an estimated 34.5 overdose cases per 10,000 persons.^8^

Many factors influence drug overdose and poisoning, such as age, lifestyle, socio-psychological burdens, and the administration route, nature, and amount of the drug.^5^,^7^,^9^ The death rate has significant associations with age and administration route.^10^ Several studies have found that the majority of poisoning cases among adults were intended, whereas those among children were accidental and occurred in those younger than 5 years old.^5^,^10^,^11^ The most commonly involved drugs were painkillers, antipsychotics, and antidepressants.^6^,^12^

In Saudi Arabia, several studies have been conducted to explore the patterns of chemical substance and drug poisoning incidence and outcomes in different regions (Riyadh, Al-Qassim, Najran, Makkah, and Jeddah).^5^ Three studies were conducted in Jeddah, in 1990,1998, and 2014.^5^ The present study focused on the prevalence of the drug overdose and poisoning, associated risk factors (gender, age and exposure circumstances), and outcomes to assess the magnitude of this health problem and to provide healthcare policy-makers with evidence to develop strategies to reduce and prevent this health problem.

## METHODS

This retrospective chart review was conducted in 2016. The authors collected and reviewed all the patient records of poisoning caused by drug overdose in January 2011–March 2016 from the Ministry of Health in Jeddah, Saudi Arabia. Cases of chemical poisoning were excluded. The data collection sheet covered patients’ age, gender, nationality, exposure circumstances, exposure route, antidotes, and outcomes.

Drugs were classified into eight main groups:

- Group-1: Analgesics, non-steroidal anti-inflammatory drugs, and muscle relaxants
- Group-2: Anti-epileptic, antipsychotic, amphetamine, anti-Parkinsonian, opioid analgesic, psychoactive, and anxiolytic drugs
- Group-3: Asthma, antihistamine, flu, and cough drugs
- Group-4: Cardiovascular, blood pressure, diabetes, anticoagulants, cholesterol-lowering, and diuretic medications
- Group-5: Antibiotic, antifungal, and antiprotozoal drugs.
- Group-6: Antispasmodic, stomach antacid, anti-vomiting, proton pumps inhibitors, and liver drugs.
- Group-7: Contraceptive pills and hormones
- Group-8: Vitamins and herbal drugs

### Statistical analysis

IBM SPSS Statistical (version 21) software was used to analyze the data. All data were non-parametric and reported as numbers and percentages. To conduct comparisons between the variables, Chi-square test was used, and the significance level was set at p-value<0.05.

## RESULTS

From January 2011 to March 2016, a total of 1474 drug-poisoning cases were reported, according to records of the Ministry of Health. More than half of these cases involved females (n= 885, 60%) and children 0–4 years old (n=764, 51.8%). More than two-thirds (n=1074, 72.9%) involved Saudis. The annual rate of drug-related poisoning cases ranged from 7.7% (through January-March 2016) to 23.9% in 2015. The case distribution by year quartile shows the highest rate (30.3%) in the first quartile (winter and spring) and relatively similar rates in the other three quartiles (second, third, and fourth) (Table-I).

**Table-I:** Demographic characteristics of the studied cases.

*Variables*	*n*	*%*
*Occurrences per year*		
2011	135	9.2
2012	238	16.1
2013	309	21.0
2014	326	22.1
2015	353	23.9
2016	113	7.7
*Time of occurrence (year quartiles)*		
First quartile (January–March)	447	30.3
Second quartile (April–June)	348	23.6
Third quartile (July–September)	314	21.3
Fourth quartile (October–December)	365	24.8
*Gender*		
Male	589	40.0
Female	885	60.0
*Age*		
0–4 years old	764	51.8
5–14 years old	129	8.8
15–24 years old	258	17.5
Older than 24 years old	323	21.9
*Nationality*		
Saudi	1,074	72.9
Non-Saudi	400	27.1

n=number of cases.

More than half of the cases were accidental (n=786, 53.3%), and less than one-third (n=419, 28.4%) were reported to two cases occurred through the inhalation route, and there were no reports of poisoning by the dermal route. Twelve cases (0.8%) involved laboratory investigation requests, and the results were positive in 10 cases. Only 2.2% of the patients (n=32) received antidotes, and no deaths were reported among the studied cases (the recovery rate was 100%) (Table-II).

**Table-II:** Medical characteristics of the studied cases.

*Variables*	*n*	*%*
*Exposure circumstances*		
Intentional	419	28.4
Accidental	786	53.3
Unknown	269	18.2
*Exposure route*		
Oral	1,466	99.5
Inhalation	2	0.1
Other	6	0.4
*Laboratory results*		
Positive	10	0.7
Negative	2	0.1
No request	1,462	99.2
*Antidote*		
No	1,442	97.8
Yes	32	2.2
*Outcomes*		
Recovery	1,474	100.0
Death	0	0.0

n=number of cases.

Analgesics were the most common (21.6%), followed by antipsychotics (7.7%). Among drug groups, most cases involved analgesics, non-steroid anti-inflammatory drugs, and muscle relaxants (n=373, 25.2%), followed by antiepileptic, antipsychotic, amphetamine, psychoactive, and anxiolytic drugs (n=229, 16.3%); antihistamine, asthma, flu, and cough drugs (n=157, 12.0%); and antibiotic, antifungal, and antiprotozoal drugs (n=74, 5.0%).

Statistically significant associations with gender (p<0.05) were present for only three drug types. Females were significantly more likely to be involved in cases with painkillers (p=0.0001), and males with asthma medications (p=0.01) and psychoactive and anxiolytic drugs (0.04) (Table-III).

**Table-III:** Distribution of drug groups by gender.

*Variables*	*Gender*			P-value*

	*Male*	*Female*	*Total*	
Antihistamines	41 (48.0%)	43 (51.2%)	84 (5.7%)	0.09
Painkillers	92 (28.8%)	228 (71.3%)	320 (21.6%)	0.001
Antiepileptics	30(38.0%)	49 (62.0%)	79 (5.3%)	0.71
Antipsychotics	41 (36.0%)	73 (64.0%)	114 (7.7%)	0.36
Diabetes drugs	4 (20.0%)	16 (80.0%)	20 (1.4%)	0.07
Blood pressure drugs	33 (50.8%)	32 (49.2%)	65 (4.4%)	0.07
Antibiotics	21 (33.3%)	42 (66.7%)	63 (4.3%)	0.30
Contraceptive pills	15 (34.9%)	28 (65.1%)	43 (2.9%)	0.49
Asthma drugs	35 (55.6%)	28 (44.4%)	63 (4.3%)	0.01
Psychoactive and anxiolytic drugs	18 (58.1%)	13 (41.9%)	31 (2.2%)	0.04
Hormones	7 (38.9%)	11 (61.1%)	18 (1.2%)	0.93
Vitamins	26 (50.0%)	26 (50.0%)	52 (3.6%)	0.13
Non-steroidal anti-inflammatory drugs	19 (48.7%)	20 (51.3%)	39 (2.6%)	0.87
Flu and cough drugs	17 (56.7%)	13 (43.3%)	30 (2.0%)	0.06
Other/unknown drugs	190(41.9%)	263(58.1%)	453(30.7%)	0.11

*p-value was calculated by Chi square test.

Regarding the association of age with drug type, statistically significant associations were found for antihistamines, painkillers, antipsychotics, contraceptive pills, asthma drugs, vitamins, and other/unknown medications (p<0.05). Cases of these drugs involved more children younger than 5 years old than other age groups (Table-IV).

**Table-IV:** Drug type by age group.

*Variables*	*Age (years)*					P-value*

	*0–4*	*5–14*	*15–24*	*≥ 25*	*Total*	
Antihistamines	67 (79.8%)	3 (3.6%)	9 (10.7%)	5 (6.0%)	84 (5.7%)	0.001
Painkillers	106 (33.1%)	36 (11.3%)	103 (32.2%)	75 (23.4%)	320 (21.6%)	0.001
Antiepileptics	37 (46.8%)	6 (7.6%)	16 (20.2%)	20 (26.3%)	79 (5.3%)	0.72
Antipsychotics	49 (43.0%)	17 (14.9%)	10 (8.8%)	38 (33.3%)	114 (7.7%)	0.001
Diabetes drugs	11 (55.0%)	1 (5.0%)	2 (10.0%)	6 (30.0%)	20 (1.4%)	0.65
Blood pressure drugs	43 (66.2%)	4 (6.2%)	7 (10.8%)	11 (16.9%)	65 (4.4%)	0.12
Antibiotics	27 (42.9%)	7 (11.1%)	11 (17.5%)	18 (28.6%)	63 (4.3%)	0.43
Contraceptive pills	36 (83.7%)	3 (7.0%)	2 (4.7%)	2 (4.7%)	43 (2.9%)	0.001
Asthma drugs	48 (76.2%)	7 (11.1%)	1 (1.6%)	7 (11.1%)	63 (4.3%)	0.001
Psychoactive and anxiolytic drugs	6 (19.4%)	0 (0.0%)	4 (12.9%)	21 (67.7%)	31 (2.2%)	0.40
Hormones	13 (72.2%)	1 (5.6%)	1 (5.6%)	3 (16.7%)	18 (1.2%)	0.34
Vitamins	44 (84.6%)	3 (5.8%)	4 (7.7%)	1 (1.9%)	52 (3.6%)	0.001
Non-steroidal anti-inflammatory drugs	19 (48.7%)	4 (10.3%)	10 (25.6%)	6 (15.4%)	39 (2.6%)	0.48
Flu and cough drugs	22 (73.3%)	1 (3.3%)	5 (16.7%)	2 (6.7%)	30 (2.0%)	0.07
Other/unknown drugs	236(68.0%)	29(8.4%)	41(11.8%)	69(19.9%)	347(23.5%)	0.001

*p-value was calculated by Chi square test.

Accidental exposure was significantly higher in cases involving antihistamines, antipsychotics, blood pressure medication, contraceptives, asthma medication, and vitamins (p<0.05). Intentional exposure was significantly higher in cases involving painkillers and psychoactive and anxiolytic drugs (p<0.05) (Table-V).

**Table-V:** Exposure circumstances by drug type.

*Variables*	*Exposure circumstances*				P-value*

	*Intentional*	*Accidental*	*Unknown*	*Total*	
Antihistamines	13 (15.5%)	64 (76.2%)	7 (8.3%)	84 (5.7%)	0.001
Painkillers	147 (45.9%)	118 (36.9%)	55 (17.2%)	320 (21.6%)	0.001
Antiepileptics	18 (22.8%)	40 (50.6%)	21 (26.6%)	79 (5.3%)	0.12
Antipsychotics	28 (24.6%)	52 (45.6%)	34 (29.8%)	114 (7.7%)	0.004
Diabetes drugs	8 (40.0%)	8 (40.0%)	4 (20.0%)	20 (1.4%)	0.43
Blood pressure drugs	14 (21.5%)	46 (70.8%)	5 (7.7%)	65 (4.4%)	0.01
Antibiotics	23 (36.5%)	28 (44.4%)	12 (19.0%)	63 (4.3%)	0.28
Contraceptive pills	5 (11.6%)	32 (74.4%)	6 (14.0%)	43 (2.9%)	0.01
Asthma drugs	6 (9.5%)	44 (69.8%)	13 (20.6%)	63 (4.3%)	0.003
Psychoactive and anxiolytic drugs	15 (48.4%)	11 (35.5%)	5 (16.1%)	31 (2.2%)	0.04
Hormones	2 (11.1%)	14 (77.8%)	2 (11.1%)	18 (1.2%)	0.11
Vitamins	4 (7.7%)	43 (82.7%)	5 (9.6%)	52 (3.6%)	0.001
Non-steroid anti-inflammatory drugs	7 (17.9%)	23 (59.0%)	9 (23.1%)	39 (2.6%)	0.32
Flu and cough drugs	7 (23.3%)	20 (66.7%)	3 (10.0%)	30 (2.0%)	0.29
Other/unknown drugs	122(29.3%)	243(30.9%)	88(32.7%)	453(30.7%)	0.60

*p-value was calculated by Chi square test.

## DISCUSSION

Recent decades have seen a revolution in the pharmaceutical industry, with numerous drugs invading the market, and increasing consumption of over-the-counter medications has been associated with rising incidence of drug-related problems, poisoning, and overdose.^1^-^3^ This study examined the pattern of drug poisoning in Jeddah, Saudi Arabia during a 5-year period from January 2011 to March 2016. Over this time, the frequency increased rapidly (lowest in 2011). Seasonally, drug poisoning was most common in the winter and spring (January-March), possibly be due to the higher number of influenza, chest infection, and allergy cases, leading to more use of over-the-counter medications with physician-prescribed medications (e.g., flu medications, cough syrup, antihistamines, antibiotics, analgesics, antipyretics). This finding conflicts with previous studies conducted in Jeddah in 2014 and Makkah in 2012 that found the most cases during the summer (June, July, August), which the authors attributed to the vacation season.^5^,^13^ A study in Malaysia found the highest incidence in March and the lowest in November and December.^14^

Regarding age groups, more than half of the cases involved children younger than 14 years old, and the majority younger than five years old. This finding is consistent with previous studies conducted in Riyadh, Jeddah, and Al-Qassim, in which children accounted for 60.8%, 44.2%, and 47.0% of cases, respectively. Several reasons could explain the increased incidence of drug poisoning among children: their curiosity and desire to explore, their mistaken belief that a drug is a candy due to its shape and color, and storage drugs in easy-to-open, non-child proof containers in easy-to-reach places without adult supervision.^5^,^11^,^13^,^15^ Studies in India, Malaysia, Al-Majmaah (Saudi Arabia), and the United States, though, showed higher frequency of drug poisoning among adults, especially young adults, than children (55.2%, 50%, 79.4%, and 53.8%, respectively).^8^,^14^,^16^,^17^

In the current study, more than half of the cases involved females (60%). Cases involving asthma, psychoactive, and anxiolytic drugs had significantly more male patients; while painkillers were more common among female patients. Similarly, higher rates among female patients have been reported in Saudi Arabia (Jeddah, Riyadh, and Al-Qassim), Malaysia, and the United States.^13^,^14^,^17^ In contrast, higher percentages of male patients have been reported in other studies in Saudi Arabia (Makkah and Al-Majmaah).^13^,^16^ Aligning with previous studies conducted in Saudi Arabia, the majority of the cases in the present study involved Saudis.^5^,^11^,^13^,^15^,^16^

Using the definition of suicidal patients as “patients who were exposed to more than one toxin and patients who came to the hospital within 1-3 hour since poisoning”^15^, less than one-third (28.4%) of the reported cases in the present study were intentional. This rate is lower than that found in Nepal (97.0%) and India (72.6%) but similar to the results of previous studies in Jeddah (26.4%) and Riyadh (25.5%), whose authors attributed these low rates to the high frequency of cases among children younger than five years old.^5^,^13^ Several studies have indicated that the majority of poisoning cases among children are accidental, while intentional poisoning among children likely indicates a child abuse or neglect.^11^,^16^

The oral route was the main route of administration in the present study, in accordance with research in Al-Qassim, Makkah, and similar locations.^11^,^13^ The most common drugs reported were analgesics, particularly Paracetamol (Panadol), followed by antipsychotic drugs and then antihistamine and antiepileptic drugs (Tegretol). Similar results were found in Al-Qassim, Makkah, Jeddah, and the United States, where analgesics and non-steroidal anti-inflammatory drugs were the most commonly reported. These drugs are classified as over-the-counter medications, are easy to get, and are available in almost every house, and many people use them with few precautions.^5^,^8^,^11^,^13^,^16^ Similarly, in Nepal, Malaysia, and India, analgesic drugs and non-steroid anti-inflammatory drugs in combination with antipsychotics and sedatives are the most commonly reportedly drugs in drug poisoning cases.^10^,^14^,^17^

Regarding treatment and outcomes, antidotes were given to only 32 cases, and all the patients involved in the poisoning cases recovered (no deaths). This death rate was lower than in similar studies in Makkah (1%), Al-Qassim (2.2%), and Nepal (3%).^9^-^11^

A greater level of knowledge about drug poisoning pattern could help in the recognition and classification of risk factors associated with this problem, enabling healthcare providers to make early detection and select appropriate interventions. There is a need for more community education programs to raise awareness of proper drug storage and use based on prescriptions from physicians, control of over-the-counter medications, and supervision of children (non-child proof containers). Additionally, workshops on drug poisoning and safety for the general public are recommended teaching first-aid measures that can be implemented immediately until hospital transfer or the arrival of medical support.

### Limitation

The main limitation of the current study is its retrospective nature, which could result in missing or incomplete information.

## CONCLUSION

This study has shown that most drug poisoning cases involved females and young children (younger than 5 years old). Most cases were accidental, and the most commonly used drugs were analgesics (Panadol), followed by antipsychotics, antihistamines, and antiepileptics (Tegretol).

### Authors’ Contributions

**SHA:** Study design, statistical analyses and shared manuscript writing.

**AHA:** Data collection, literature search, validation, and coding, shared in writing introduction, discussion and gathered references.

**FMF:** Data entry, validation, and coding, shared in writing introduction, discussion and literature search.

**MAGE:** Data collection, data entry, validation, and coding, shared in writing introduction and literature search.

**JB:** Logistics, interpretation and writing of results and discussion, review and final approval of manuscript.
